# The safety and efficacy of laparoscopic retrograde appendicectomy, base-to-tip approach

**DOI:** 10.3389/fsurg.2023.1256256

**Published:** 2023-09-11

**Authors:** Ara Ko, Perry Lindsay, Julian Choi

**Affiliations:** ^1^ Department of Surgery, Western Health, St Albans, VIC, Australia; ^2^ School of Medicine, Monash University, Campus Centre, Clayton, VIC, Australia; ^3^ General Surgery & Gastroenterology Clinical Institute, Epworth Richmond, Richmond, VIC, Australia

**Keywords:** appendicitis, retrograde appendicectomy, appendicectomy methods, surgical management of appendicitis, base to tip approach

## Abstract

**Background:**

Laparoscopic appendicectomy is one of the most frequently performed surgical procedures worldwide. There is limited evidence evaluating the role and safety of laparoscopic retrograde appendicectomy (LRA), base to tip approach, compared to standard laparoscopic antegrade appendicectomy (LAA), tip to base approach. This study aims to assess the safety of LRA compared to LAA in terms of intra-abdominal collection (IAC) rate and using Sunshine Appendicitis Grading System (SAGS).

**Methods:**

Records of two-hundred and seventy-three patients undergoing laparoscopic appendicectomy by LAA and LRA approaches were analysed. The severity of appendicitis was rated using a standardised Sunshine Appendicitis Grading System (SAGS) score intra-operatively. The primary outcome measure was the occurrence of an intra-abdominal collection, and secondary measures were procedure time, post-operative length of stay and other complications.

**Results:**

Of the two-hundred and seventy-three patients, there were two patients who developed an intra-abdominal collection. Both patients were in the LAA group with SAGS IV scores. Between SAGS IV patients, Chi-squared *p* value of 0.6691. Therefore, there was no statically significant difference in the intra-abdominal collection (IAC) rate between LAA and LRA groups from this study.

**Conclusions:**

The current study has shown that laparoscopic retrograde appendicectomy (LRA) does not increase risk of intra-abdominal collection compared to laparoscopic antegrade appendicectomy (LAA) within the limit of this study.

## Introduction

Appendicitis is one of the most common causes of abdominal pain, with an estimated lifetime risk of 7%–8% globally (1, 2). Surgical approaches to appendicectomy have developed significantly since McBurney first described an open approach in 1894, with laparoscopic appendicectomy first described by Semm in 1983 (3, 4). The laparoscopic method is now the standard treatment for acute appendicitis with reduced rate of wound site infection and shortened hospital length of stay (1, 5).

In Laparoscopic Antegrade Appendicectomy (LAA), the tip of appendix is first identified and its mesentery is serially divided using diathermy and clips toward the base of appendix, which is divided after securing with applying an endo-loop (4). This can be challenging when the tip of the appendix is not easily accessible (6).

Another approach is the laparoscopic retrograde appendicectomy (LRA), first described by Motson and Kelly in 2002 (7), in which the base of the appendix is first identified and divided prior to mobilisation of the appendix to its tip. Given the base of the appendix is divided prior to controlling the stump, there is perceived risk of faecal contamination and subsequent development of intra-abdominal collection (IAC). However, there is lack of evidence in efficacy and safety of LRA compared to LAA especially in terms of risk of IAC. Thus, this study aims to review the surgical technique and utility of LRA and examine the results of the LRA compared with LAA including; the risk of IAC, length of inpatient stay post procedure, intra-operative time for procedure and other complications.

The Sunshine Appendicitis Grading System (SAGS) score is an intraoperative grading system for acute appendicitis, first described in 2015 by F. Reid et al. (8), which correlates severity of disease with the risk of post-operative intra-abdominal collection.

## Method

### Data collection

This retrospective observational study was designed to evaluate the rate of post-operative complications following the LRA in comparison to the LAA according to their SAGS classifications. Medical records of two hundred seventy-two patients who underwent laparoscopic appendicectomy performed by a single surgeon at one institution between January 2014 and July 2021 were reviewed including; type of surgical approach including antegrade vs. retrograde appendicectomy, Sunshine Appendicitis Grading System (SAGS) score (Table 1), operative time, post-operative length of stay, post-operative complications and readmissions. Primary outcome was the rate of intra-abdominal collection (IAC), diagnosed on computed tomography (CT) or by ultrasound scanning in those with clinical suspicion of IAC. Secondary outcome measures include procedural time, readmission rate and other post-operative complications. Patients who underwent appendicectomy in conjunction with another procedure were included in this study. Ethics approval is achieved.

**Table 1:** The SAGS score (9).

SAGS Score	Intra-operative findings
0	No appendicitis
1	Simple appendicitis (any of the following): i. Injected appendix ii. Thickened appendix iii. Serous free fluid
2	Purulent appendicitis (any of the following): i. Pus localised to right iliac fossa ii. Right paracolic gutter iii. Pelvis
3	Purulent appendicitis with 4 quadrant contaminations
4	Perforated appendix (any of the following): i. Free faecolith, faeces ii. Faecal staining iii. Visible hole in appendix

### Operative technique of LRA

In this study, LRA is performed in patients with retro-colic, retro-iliac and pelvic appendicitis. After an open Hassan port entry, pneumoperitoneum with CO_2_ insufflation up to 12–15 mmHg is established. Following insertion of two 5 mm ports into the right and the left iliac fossa under direct vision the base of the appendix is identified. A small peritoneal window is created in the mesoappendix adjacent to the base and is gently compressed using a non-tooth grasper to displace any faecolith away from the base. The appendiceal stump is cut 1–2 cm from the base with laparoscopic scissors and a PDS Endoloop (Ethicon Endo-Surgery, Johnson and Johnson, Cincinnati, OH, USA) is placed on both cut ends of the appendix. The remaining appendix is mobilised by dividing the mesoappendix with diathermy and clips or with a 5 mm LigaSure Maryland (Metronic, Dublin, Ireland). When the base of appendix is friable or necrotic, the caecum is mobilised and *en bloc* caecectomy is performed using a laparoscopic stapling device. The specimen is retrieved with an EndoCatch bag (Metronic, Dublin, Ireland) after ensuring haemostasis.

### Statistical analysis

Collected data was analysed using excel spreadsheets and statistical programs R [R Core Team (2022). R: A language and environment for statistical computing. R Foundation for Statistical Computing, Vienna, Austria. URL http://www.R-project.org/.] and STATA (StataCorp. 2021. *Stata Statistical Software: Release 17*. College Station, TX: StataCorp LLC.), with Chi-square test, Fisher's exact test and t test as required.

## Results

### Patient demographics

A total of 273 patients were included. There were 134 male and 139 female patients, aged between 9 and 84 years with a mean age of 35. 47% of patients had SAGS grade I appendicitis. The rate of follow up was 87% which was conducted via surgeon's private rooms within 30 days post discharge. Patient demographics are shown in the Table 2.

**Table 2:** Patient demographics.

		Number	%	Mean	Median	Range
Gender	Male	134	49			
	Female	139	51			
Age				35	32	9–84
SAGS	0	55	20.1			
	I	128	46.9			
	II	70	25.6			
	III	2	0.7			
	IV	18	6.6			

### Severity of disease/prognosis

**Figure 1:**
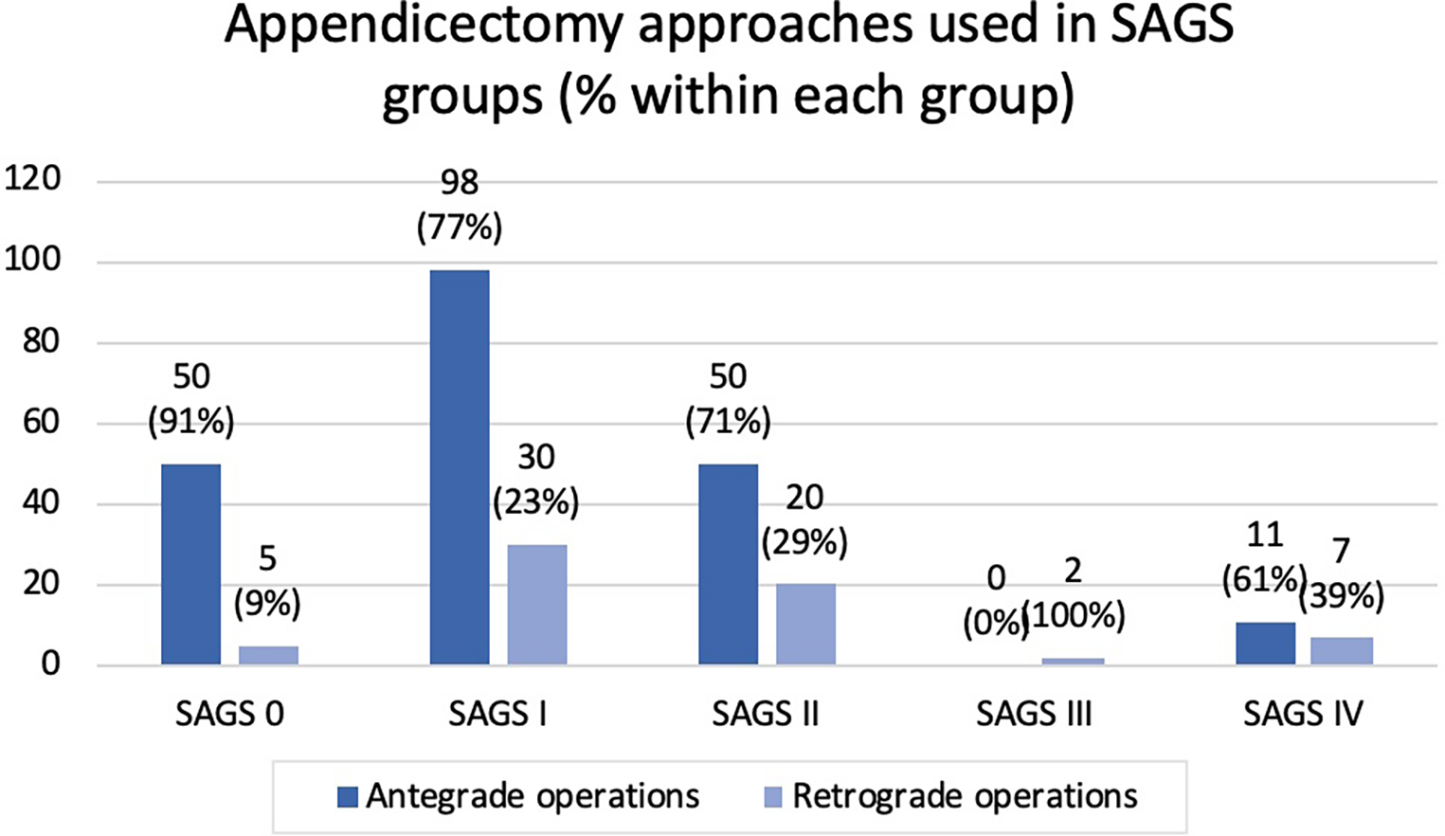
Appendicectomy approaches used in different SAGS groups.

With increasing SAGS score and appendicitis severity the proportion of LRA utilised increased, as shown in the Figure 1. This was statistically analysed using a chi-squared test demonstrating a *p* value of 0.003, therefore this difference was shown to be statistically significant.

### Intra-abdominal collection occurrence analysis

209 patients underwent the LAA and 64 the LRA. Out of 273 patients, there were only two patients who developed IAC, who both underwent LAA approach with perforated appendicitis (SAGS IV). There was no IAC for SAGS score 3 and below. Overall, there was no statistically significant difference in occurrence of IAC between patients having LAA and LRA appendicectomy. Chi-squared test *p* value of the analysis was 0.43 with power of 0.195. The overall rate of IAC for SAGS IV was 11% (2/18); LAA was 18% (2/11) and none in LRA (0/7), with Fisher's exact test *p* value of 0.50. Therefore, there was no statistically significant difference in post-operative IAC rate between LAA and LRA overall and amongst SAGS IV groups from this study. Both patients with IAC were managed non-operatively, one requiring percutaneous radiologically guided drainage.

### Secondary outcome measures

The mean procedural times for LAA and LRA were 34.85 min and 40.92 min respectively [*p* = 0.002, CI (−9.29, −2.86)], indicating a statistically significant longer procedural time for LRA. The mean length of stay for LAA and LRA patients were 1.6 days and 1.77 days respectively [*p* = 0.208, CI (−0.57, 0.12)], which was not statistically significant. Three patients were re-admitted within 30 days for post-operative pain, one with pulmonary embolism and two with post-operative ileus. The two patients who suffered ileus both underwent LAA appendicectomies with SAGS score of 4.

## Discussion

In difficult appendicectomies, LRA (base to tip approach) has been described as a valuable alternative to LAA (tip to base approach) (6, 7, 9). However, there has been limited literature on the safety and efficacy of LRA given there has not been a standardised way of classifying the intraoperative severity of appendicitis. This is the first study to compare the risk of post-operative complications for LRA with the LAA, using a classification for severity of acute appendicitis such as SAGS (8, 10).

As demonstrated in Figure 1, LRA was used in preference to LAA as severity of appendicitis increases in SAGS scores. Although there was a statistically significant increase in operation time for LRA, this six minute difference is unlikely to be clinically relevant. In fact, LRA was used in technically more challenging cases, where the tip of the appendix is not easily identifiable. Thus, LRA may have decreased overall procedural time, rate of open conversion and need for right hemicolectomy. Despite LRA being utilised for more severe appendicitis there were no intra-abdominal collections found following LRA. However, this study is limited with low power due to the low rate of IAC and small number of patients with SAGS score III and IV. Although LAA or LRA did not affect post-operative IAC rate, rate of IAC increased with SAGS scores as previously shown by Reid et al. (8) This study is also limited due to the nature of the retrospective study design, small sample size and in that data was solely collected from cases performed by a single operator from one institution.

There was no significant difference in post-operative admission length or secondary outcome measures such as readmission and post-operative ileus. This suggests that there was no increase in immediate post-operative complications or increased cost associated with LRA.

Given the base of the appendix is divided prior to controlling the stump, there is perceived risk of faecal contamination and subsequent development of IAC. More recently, Mathews in 2020 further developed LRA method where a window is dissected in the mesoappendix and the base is initially divided with diathermy instead of cut using a pair of scissors (7, 9). As described by Matthews, there are various methods of LRA which may alter the rate of post-operative complications (9). For example, the base of the appendix can be stapled if concerned about the integrity of the appendiceal stump (6).

There are a number of advantages of LRA over LAA: LRA is relatively easy to perform as long as the appendiceal stump can be identified; it is effective and efficient in difficult appendicectomies such as retro-caecal, retro-ileal and pelvic appendicitis; and it is not associated with higher rates of complications including IAC.

## Conclusion

In conclusion, LRA (Laparoscopic retrograde appendicectomy, base to tip) is a safe alternative to LAA (Laparoscopic antegrade appendicectomy, tip to base) in surgically challenging appendicitis without increased risk of complication within the limit of this study.

## Data Availability

The raw data supporting the conclusions of this article will be made available by the authors, without undue reservation.
